# Characterization of a Novel Plasmid Type and Various Genetic Contexts of *bla*_OXA-58_ in *Acinetobacter* spp. from Multiple Cities in China

**DOI:** 10.1371/journal.pone.0084680

**Published:** 2014-01-06

**Authors:** Yiqi Fu, Jingjin Jiang, Hua Zhou, Yan Jiang, Ying Fu, Yunsong Yu, Jianying Zhou

**Affiliations:** 1 Department of Respiratory Diseases, The First Affiliated Hospital, College of Medicine, Zhejiang University, Hangzhou, China; 2 Department of VIP, The First Affiliated Hospital, College of Medicine, Zhejiang University, Hangzhou, China; 3 Department of Infectious Diseases, Sir Run Run Shaw Hospital, College of Medicine, Zhejiang University, Hangzhou, China; University of Edinburgh, United Kingdom

## Abstract

**Background/Objective:**

Several studies have described the epidemiological distribution of *bla*_OXA-58_-harboring *Acinetobacter baumannii* in China. However, there is limited data concerning the replicon types of *bla*_OXA-58_-carrying plasmids and the genetic context surrounding *bla*_OXA-58_ in *Acinetobacter* spp. in China.

**Methodology/Principal Findings:**

Twelve non-duplicated *bla*_OXA-58_-harboring *Acinetobacter* spp. isolates were collected from six hospitals in five different cities between 2005 and 2010. The molecular epidemiology of the isolates was carried out using PFGE and multilocus sequence typing. Carbapenemase-encoding genes and plasmid replicase genes were identified by PCR. The genetic location of *bla*_OXA-58_ was analyzed using S1-nuclease method. Plasmid conjugation and electrotransformation were performed to evaluate the transferability of *bla*_OXA-58_-harboring plasmids. The genetic structure surrounding *bla*_OXA-58_ was determined by cloning experiments. The twelve isolates included two *Acinetobacter pittii* isolates (belong to one pulsotype), three *Acinetobacter nosocomialis* isolates (belong to two pulsotypes) and seven *Acinetobacter baumannii* isolates (belong to two pulsotypes/sequence types). *A. baumannii* ST91 was found to be a potential multidrug resistant risk clone carrying both *bla*_OXA-58_ and *bla*_OXA-23_. *bla*_OXA-58_ located on plasmids varied from ca. 52 kb to ca. 143 kb. All plasmids can be electrotransformed to *A. baumannii* recipient, but were untypeable by the current replicon typing scheme. A novel plasmid replicase named *rep*Aci10 was identified in *bla*_OXA-58_-harboring plasmids of two *A*. *pittii* isolates, three *A*. *nosocomialis* isolates and two *A. baumannii* isolates. Four kinds of genetic contexts of *bla*_OXA-58_ were identified. The transformants of plasmids with structure of IS*6* family insertion sequence (IS*Our1*, IS*1008* or IS*15*)-ΔIS*Aba3*-like element-*bla*_OXA-58_ displayed carbapenem nonsusceptible, while others with structure of intact IS*Aba3*-like element-*bla*_OXA-58_ were carbapenem susceptible.

**Conclusion:**

The study revealed the unique features of *bla*_OXA-58_-carrying plasmids in *Acinetobacter* spp. in China, which were different from that of *Acinetobacter* spp. found in European countries. The diversity of the genetic contexts of *bla*_OXA-58_ contributed to various antibiotics resistance profiles.

## Introduction

Members of the genus *Acinetobacter* are significant nosocomial pathogens. *Acinetobacter baumannii* and its two close relatives, *Acinetobacter pittii* and *Acinetobacter nosocomialis* account for the majority of *Acinetobacter* infections [1]. A number of reports have detailed the significant increase in resistance of *Acinetobacter* spp. to conventional antibiotics, including carbapenems, the main therapeutic alternative against multidrug resistant *Acinetobacter* infections [2].

The worldwide emergence of carbapenem resistant *Acinetobacter* may be attributed to the spread of some risk resistant clones and the horizontal transmission of carbapenemase genes [1], [3]. Carbapenem-hydrolyzing class D β-lactamases (CHDLs) are the most concerning carbapenem resistant determinants in *Acinetobacter* spp [1]. OXA-58 is a widely spread CHDL that has been reported in *Acinetobacter* spp. from Europe [4], Argentina [5], Australia [6], the United States [7] and many Asian countries [8]. Though OXA-58 shows only low carbapenem-hydrolyzing activity *in vitro*, the insertion sequence upstream of *bla*_OXA-58_ enhances its transcription greatly and mediates resistance to carbapenems [9]–[11].

*bla*_OXA-58_ exists not only in *A. baumannii*, but also in *A. pittii* [12], *A. nosocomialis* [11], *Acinetobacter radioresistens* [13], *Acinetobacter junii* [6], and *Acinetobacter* phenon 6/ct13TU [14]. *bla*_OXA-58_ is usually plasmid-borne, which may explain its wide dissemination. It has been reported that OXA-58 producing *A. baumannii* from European countries are associated with carriage of plasmid replicase gene *rep*Aci1 [15]. However, little is known about the replicon types of *bla*_OXA-58_-carrying plasmids in *A. baumannii* and non-*baumannii Acinetobacter* spp. outside of Europe.

*bla*_OXA-58_ is the second most frequently identified CHDL in *A. baumannii* in China. However, the current data is limited to simple epidemiological distribution [16], [17]. In this study, we detailed characterized the genetic contexts surrounding *bla*_OXA-58_ and the replicon typing of the *bla*_OXA-58_-carrying plasmids in *Acinetobacter* spp. isolates from multiple cities in China.

## Materials and Methods

### Bacterial Strains and Antimicrobial Susceptibility Testing

Twelve non-duplicated *bla*_OXA-58_-harboring *Acinetobacter* spp. isolates collected from six hospitals in five different cities in China between 2005 and 2010 were analyzed in this study (Table 1). The genomic species identification was performed by sequence analysis of the 16S-23S rRNA intergenic spacer region [18].

**Table 1:** Basic information, epidemiological features and resistant genes of *Acinetobacter* spp. included in this study^a^.

Strain	Species	Hospital (Cities)	Year	PFGEtype	ST	allelic profiles^b^	*bla*_OXA_ genes	ESBLgenes
AP04	*A. pittii*	HZ (Hangzhou)	2009	A	ND	–	*bla*_OXA-58_	Neg
AP25	*A. pittii*	TZ (Taizhou)	2009	A	ND	–	*bla*_OXA-58_	Neg
AN113	*A. nosocomialis*	WZ (Wenzhou)	2009	B	ND	–	*bla*_OXA-58_	Neg
AN116	*A. nosocomialis*	WZ (Wenzhou)	2009	B	ND	–	*bla*_OXA-58_	Neg
AN119	*A. nosocomialis*	WZ (Wenzhou)	2009	C	ND	–	*bla*_OXA-58_	Neg
WA3	*A. baumannii*	WHC (Wuhan)	2008	E	363	51-54-49-11-48-25-4	*bla*_OXA-58_, *bla*_OXA-51_	*bla*_PER-1_
WA8	*A. baumannii*	WHC (Wuhan)	2008	E	363	51-54-49-11-48-25-4	*bla*_OXA-58_, *bla*_OXA-51_	*bla*_PER-1_
WH8144	*A. baumannii*	WH (Wuhan)	2010	D	91	22-15-13-12-4-62-2	*bla*_OXA-58_, *bla*_OXA-23_, *bla*_OXA-51_	Neg
JH01	*A. baumannii*	JH (Jinhua)	2005	D	91	22-15-13-12-4-62-2	*bla*_OXA-58_, *bla*_OXA-23_, *bla*_OXA-51_	Neg
JH02	*A. baumannii*	JH (Jinhua)	2005	D	91	22-15-13-12-4-62-2	*bla*_OXA-58_, *bla*_OXA-23_, *bla*_OXA-51_	Neg
AB212	*A. baumannii*	JH (Jinhua)	2009	D	91	22-15-13-12-4-62-2	*bla*_OXA-58_, *bla*_OXA-23_, *bla*_OXA-51_	Neg
AB222	*A. baumannii*	JH (Jinhua)	2009	D	91	22-15-13-12-4-62-2	*bla*_OXA-58_, *bla*_OXA-23_, *bla*_OXA-51_	Neg
LS0148	*A. baumannii*	LS (Lishui)	2005	ND	20	1-15-13-12-4-12-2	*bla*_OXA-51_	Neg

^a^ Abbreviations: HZ, Hangzhou First hospital; TZ, Taizhou Hospital; WZ, The First Affiliated Hospital of Wenzhou Medical College; WHC, Wuhan Children Hospital; WH, Wuhan Tongji Hospital; JH, Jinhua Center Hospital; LS, Lishui People Hospital; ND: not defined; Neg: negative; Pos: positive.

^b^ Seven loci in the order of *gltA*-*gyrB*-*gdhB*-*recA*-*cpn60*-*gpi*-*rpoD*.

Imipenem and ticarcillin-susceptible clinical *A. baumannii* strain LS0148 (imipenem MIC, 0.5 mg/L; ticarcillin MIC, 16 mg/L), deposited in our laboratory, was used as the recipient for plasmid electrotransformation (Table 1). A colistin-resistant mutant strain of *A. baumannii* LS0148 (colistin MIC, 64 mg/L) was used as the recipient for plasmid conjugation.

MICs were determined by the agar dilution method. Interpretation of the results was in accordance with the CLSI 2013 criteria.

All isolates present in this study were stored in the Department of Microbiology, the First Affiliated Hospital, College of Medicine, Zhejiang University. We obtained an exempt status from the Institutional Review Board of the First Affiliated Hospital, College of Medicine, Zhejiang University to use these strains to perform all experiments in this study.

### PCR Experiments for the Resistance Genes

PCR assays for the presence of carbapenemase encoding genes (*bla*_OXA-51_-like, *bla*_OXA-58_-like, *bla*_OXA-23_-like, *bla*_OXA-40_-like, *bla*_OXA-143_, *bla*_IMP_, *bla*_VIM_, *bla*_SIM_ and *bla*_NDM_) and ESBL genes (*bla*_PER_ and *bla*_SHV_) were performed as previously reported [19]–[21].

### Pulsed-field Gel Electrophoresis and Multilocus Sequence Typing Analysis

The genetic relationship of the isolates was evaluated by pulsed-field gel electrophoresis (PFGE) and multilocus sequence typing (MLST). The results of PFGE were interpreted as Tenover et al. recommended [22]. MLST was carried out using the scheme developed by Bartual et al. with some modifications to the primers of the alleles of *gyrB* and *rpoD* as we previously reported [23], [24].

### Plasmid Conjugation and Electrotransformation

Plasmid conjugations were performed between OXA-58 producing *Acinetobacter* spp. as donors and a colistin-resistant mutant strain of *A. baumannii* LS0148 as the recipient. The transconjugants were selected on MH agar plates containing ticarcillin (100 mg/L) and colistin (10 mg/L).

The electrical pulse setting of plasmid electrotransformation was 1.8 kV, 25 µF, 200 Ω with Bio-Rad GenePulser Xcell system (Bio-Rad, Shanghai, China). *A. baumannii* strain LS0148 was used as the recipient. The transformants were selected on MH agar plates containing ticarcillin (100 mg/L).

### S1 Nuclease-based Plasmid Analysis

The plasmid size and the location of *bla*_OXA-58_ were analyzed using the S1 nuclease-PFGE method as previously reported [25]. The bacterial-imbedded gel slices were incubated with 10 U S1 nuclease (Takara, Dalian, China) for 40 minutes in 37°C water bath. The digestion products were separated by PFGE using Bio-Rad CHEF Mapper XA system (Bio-Rad, Shanghai, China) with switch times of 2.16S to 63.8S for 18 hours.

The separated DNA was transferred to a positive charged Nylon membrane (Millipore, Shanghai, China) and hybridized with a digoxigenin-labled *bla*_OXA-58_ probe. The detection of hybrids was performed using enzyme immunoassay and NBT/BCIP coloration according to the manufacturer’s instruction (Roche, Shanghai, China).

### PCR-based Plasmid Replicon Typing

The plasmid replicase genes were detected by multiplex PCR scheme developed by Bertini et al [26]. The novel replicase gene *rep*Aci10 was detected by a single PCR with primers designed in this study (Forward primer: 5′-TAGGACGTCAAGCATCTTA-3′; backward primer: 5′-TCGCTATCAAGAAGATCAC-3′).

### Cloning Experiments

The genetic contexts of *bla*_OXA-58_ were determined by cloning and sequencing experiments. The plasmids or total DNA were digested by EcoRI or SacI. The digested fragments were inserted into corresponding sites of pET28a, and the ligation mixture was used for transformation. Transformants were selected on MH agar containing ampicillin 50 mg/L and kanamycin 50 mg/L. The *bla*_OXA-58_-containing inserts were fully or partially sequenced to obtain the context of *bla*_OXA-58_.

### Nucleotide Sequence Accession Numbers

The novel insertion sequence IS*Aba20* has been submitted to the IS Finder Database (http://www-is.biotoul.fr/). The nucleotide sequences surrounding *bla*_OXA-58_ of AN119, AP04, WA3 and WH8144 are deposited in the GenBank database under accession no. JQ241789 to JQ241792 respectively.

## Results

### Species Identification and Antimicrobial Susceptibility Profiles

The 12 OXA-58-producing *Acinetobacter* spp. isolates were assigned to three genomic species: *A. baumannii* (seven isolates), *A. nosocomialis* (three isolates) and *A. pittii* (two isolates), and showed various resistance profiles (Table 1 and 2). The five non-*baumanii Acinetobacter* displayed imipenem and meropenem susceptible. On the contrary, all of the *A. baumannii* isolates were imipenem and meropenem resistance. In general, the *A. baumannii* isolates were more frequently resistant to broad-spectrum cephalosporins, ampicillin/sulbactam, aminoglycosides, ciprofloxacin and minocycline than the five non-*baumanii Acinetobacter*.

**Table 2:** The sizes and replicon types of *bla*_OXA-58_-harboring plasmids, genetic contexts of *bla*_OXA-58_, and MICs (mg/L) of represented strains^a^.

Isolates^b^	Plasmidsize (kb)	*rep* genegroup^c^	Geneticcontextsof *bla*_OXA-58_ d	IPM	MEM	FEP	CAZ	CTX	SAM	TZP	TIC	GEN	AMK	MIN	CIP
AP04	93	*aci10*	A	0.5	0.5	4	4	16	4	32	>256	64	2	<0.125	<0.125
AP25	52	*aci10*	A	1	0.5	2	4	16	2	16	>256	2	2	<0.125	<0.125
AN113	143	*aci10*	A	0.5	1	4	4	16	1	16	>256	>256	4	4	0.5
AN116	143	*aci10*	A	1	2	16	8	32	16	128	>256	>256	4	4	0.25
AN119	76	*aci10*	D	4	2	4	8	16	4	64	>256	128	4	0.5	0.25
WA3	101	*aci10*	C	>32	8	256	>256	>256	64	256	>256	64	16	<0.125	<0.125
WH8144	55	–	B	>32	>32	128	32	64	128	>256	>256	>256	256	64	64
JH01	55	GR8	B	>32	>32	256	128	256	128	>256	>256	>256	>256	64	16
AB212	55	–	B	>32	>32	32	64	128	32	>256	>256	>256	>256	32	16
LS0148	–	[GR3, GR7]	–	0.5	0.5	2	4	16	2	16	16	1	2	8	16
TAP04	93	*aci10*, [GR3, GR7]	A	2	2	4	8	16	4	64	>256	16	2	8	32
TAP25	52	*aci10*, [GR3, GR7]	A	2	1	4	8	16	4	64	>256	4	4	8	32
TAN113	143	*aci10*, [GR3, GR7]	A	1	1	4	8	16	2	32	>256	>256	4	4	32
TAN116	143	*aci10*, [GR3, GR7]	A	1	2	4	4	16	2	16	>256	>256	4	4	32
TAN119	76	*aci10*, [GR3, GR7]	D	16	16	4	8	16	16	256	>256	64	4	8	32
TWA3	101	*aci10*, [GR3, GR7]	C	16	8	4	8	16	16	256	>256	1	2	8	16
TWH8144	55	[GR3, GR7]	B	8	4	4	8	16	8	128	>256	256	128	8	16
TJH01	55	[GR3, GR7]	B	8	4	4	8	16	8	128	>256	256	128	8	16
TAB212	55	[GR3, GR7]	B	16	4	4	8	16	8	256	>256	256	128	8	16

^a^ IPM, imipenem; MEM, meropenem; FEP, cefepime; CAZ, ceftazidime; CTX, cefotaxime; SAM, ampicillin/sulbactam; TZP, piperacillin/tazobactam; TIC, ticarcillin; GEN, gentamicin; AMK, amikacin; MIN, minocycline; CIP, ciprofloxacin.

^b^ The isolates with names starting with alphabet T- were transformants;

^c^ Brackets indicate that GR3 and GR7 replicases are present in the recipient strain LS0148.

^d^ The alphabet corresponded to four kinds of genetic structure displayed in Figure 1.

### Molecular Epidemiology of the OXA-58-producing *Acinetobacter* spp.

PFGE identified five pulsotypes among the 12 OXA-58-producing *Acinetobacter* spp. isolates (Table. 1). Two *A*. *pittii* isolates from different hospitals showed a same pulsotype. Three *A*. *nosocomialis* isolates from a single hospital belonged to two pulsotypes. Seven *A. baumannii* isolates were divided into two pulsotypes, corresponding to two sequence types (ST91 and ST363). *A. baumannii* ST91 were identified from two hospitals in different cities (Wuhan and Jinhua). Moreover, ST91 were detected in *A. baumannii* collected from Jinhua Center Hospital in 2005 and 2009, implying probable endemic in this hospital.

### Distribution of Resistance Genes

The *A*. *pittii* and *A*. *nosocomialis* were negative for other carbapenemase genes and ESBLs. Intrinsic *bla*_OXA-51_ was detected in all *A. baumannii* isolates. All *A. baumannii* ST91 isolates carried another CHDL gene *bla*_OXA-23_. *bla*_PER-1_ was detected in WA3 and WA8 (Table 1).

### The Plasmid Localization of *bla*_OXA-58_

The *bla*_OXA-58_-probe hybridized with plasmid bands of different sizes, from ca. 52 kb to 143 kb. Isolates with same pulsotypes generally possessed same plasmid location of *bla*_OXA-58_, except AP04 and AP25 (Table 2).

### The Transferability of *bla*_OXA-58_-carrying Plasmids

While plasmid conjugation ultimately failed, *bla*_OXA-58_-carrying plasmids were successfully electro-transferred from all *Acinetobacter* spp. isolates to the recipient strain.

PCR detection of transformants found *bla*_PER-1_ and *bla*_OXA-23_ were not co-transferred with *bla*_OXA-58_, suggesting these genes are not colocalized on a single plasmid.

The results of antimicrobial susceptibility testing are presented in Table 2. Electrotransformation of *bla*_OXA-58_-harboring plasmids into recipient strain LS0148 resulted in high resistance to ticarcillin (>256 mg/L) and increased MICs of imipenem and meropenem (2 to 32 folds), but transformants retained similar MICs of cefepime, ceftazidime and cefotaxime when compared with that of the original LS0148 strain. The transformants of *A. nosocomialis* AN119 and all *A. baumannii* displayed higher MICs of imipenem and meropenem than transformants of *A. pittii* isolates and remaining *A. nosocomialis* isolates. Transformants TWH8144, TJH01, TAB212 also showed gentamicin and amikacin resistance, implying potential aminoglycosides resistant determinants are colocalized with *bla*_OXA-58_ on the same plasmid.

### Identification a Novel Plasmid Replicase Gene

**Figure 1:**
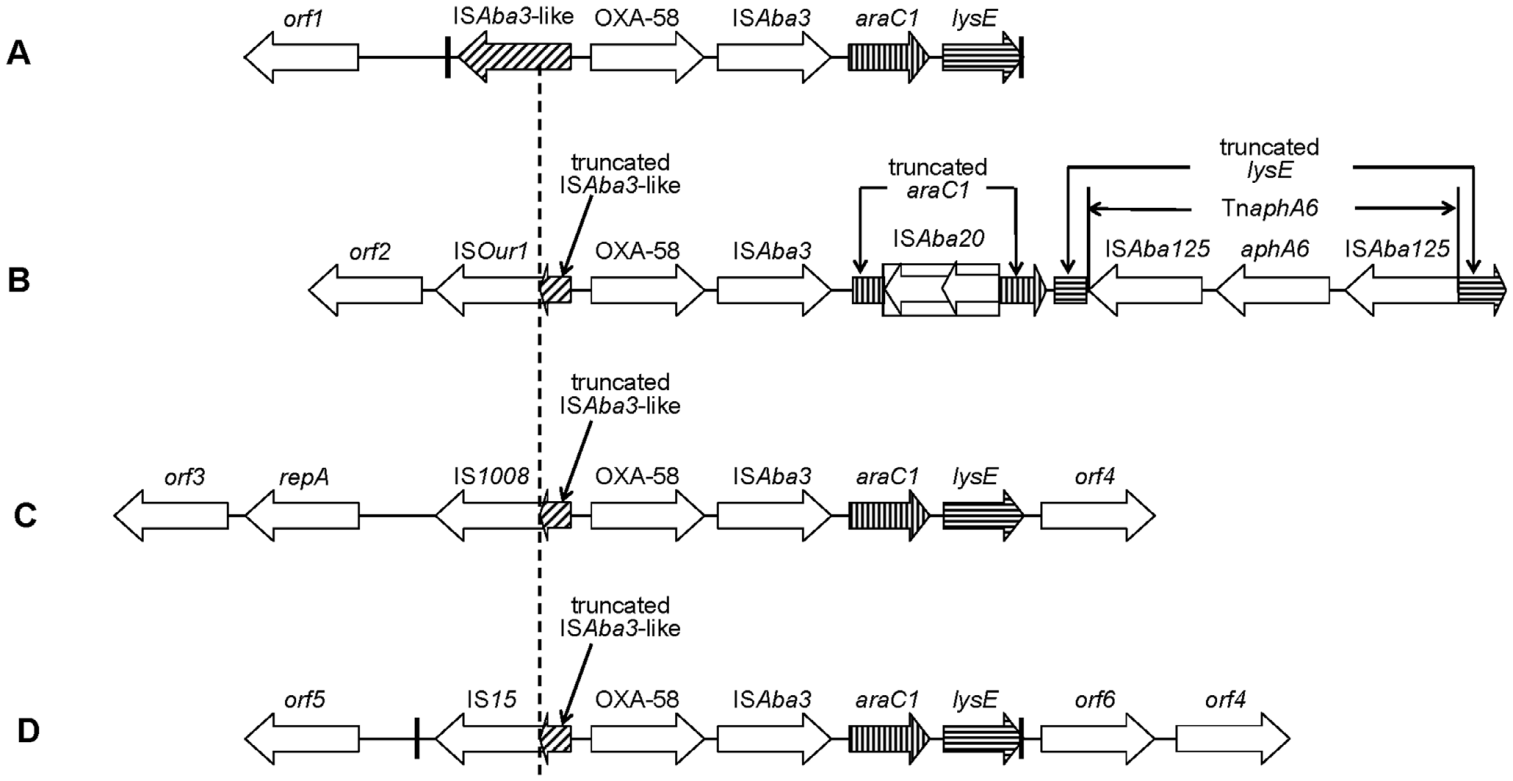
Schematic map of the genetic contexts of *bla*_OXA-58_. Structure A (AP04, AP25, AN113 and AN116); Structure B (WH8144, JH01, JH02, AB212 and AB222); Structure C (WA3 and WA8); Structure D (AN119). The dash line indicates the truncated positions of IS*Aba3*-like element. The thick vertical lines indicate the Re27 recombination points. The location and orientation of primers are indicated by arrows and numbers, being consistent with Table 2. *orf1*, DNA-binding response regulator gene; *orf2*, putative exodeoxyribonuclease VII large subunit gene; *orf3*, ParA family protein gene; *orf4*, putative inner membrane protein gene; *orf5*, putative chromate transporter gene; *orf6*, putative cytoplasmic protein gene. GenBank accession No.: A, JQ241790; B, JQ241792; C, JQ241791; D, JQ241789. The figure is not to scale.

Further investigation of the *bla*_OXA-58_-containing clone fragment of strain WA3 identified a novel plasmid replication protein gene (Figure. 1). This replication protein belonged to Rep-3 superfamily group. It shared similarity with two replication proteins deposited in GenBank database: *Acinetobacter* sp. RUH2624 (ZP_05826577; 100% amino acid identity) and *A. radioresistens* SH164 (ZP_06073941; 73% amino acid identity). We have designated this novel replicase gene as *rep*Aci10 herein. Of the available *A. baumannii* replicase genes in the current replicon typing scheme [26], *rep*Aci5 was most similar to *rep*Aci10 (66% nucleotide identity). Therefore, *rep*Aci10 should be assigned as a novel homolog group (GR20). No iteron was identified upstream of *rep*Aci10.

Using the current PCR-based replicon typing scheme of *A. baumannii* [26], only GR8 was detected in strain JH01 and JH02 from the 12 OXA-58 producing *Acinetobacter* spp. (Table 2). GR3 and GR7 are the intrinsic plasmid *rep* genes of recipient strain LS0148. No other replicase genes were detected in the transformants except for the intrinsic plasmid replicase genes of LS0148 (GR3 and GR7), suggesting the *bla*_OXA-58_-carrying plasmids do not belong to any previously known replicon group. The novel replicase gene *rep*Aci10 was detected in *A. pittii* (AP04, AP25), *A. nosocomialis* (AN113, AN116 and AN119), *A. baumannii* (WA3, WA8) and their transformants (Table 2).

### Genetic Contexts of *bla*_OXA-58_

Four kinds of genetic contexts of *bla*_OXA-58_ were identified among 12 *Acinetobacter* spp. (Figure. 1). Structure A included two *A. pittii* isolates (AP04, AP25) and two *A. nosocomialis* isolates (AN113 and AN116). Structure B encompassed all *A. baumannii* ST91 isolates of WH8144, JH01, JH02, AB212 and AB222. Structure C encompassed *A. baumannii* isolates WA3 and WA8. Structure D included *A. nosocomialis* isolate AN119. The most notable difference was the IS elements located upstream of *bla*_OXA-58_. In structure A, an intact IS*Aba3*-like element was exclusively present upstream of *bla*_OXA-58_. However, in structure B, C and D, the IS*Aba3*-like element upstream of *bla*_OXA-58_ was truncated at a same position (58 bp downstream of the start codon of the transposase gene of IS*Aba3*-like element) by IS*Our1*, IS*1008* and IS*15* respectively. All of the latter three IS elements belong to the IS*6* family. The transformants of the plasmids with the structure of IS*6* family-ΔIS*Aba3*-like-*bla*_OXA58_ displayed a much higher increase in imipenem MICs (16–32 folds) than those with intact IS*Aba3*-like-*bla*_OXA58_ (2–4 folds) (Table 2).

IS*Aba3*, *araC1* (putative transcriptional regulator gene) and *lysE* (putative threonine efflux protein gene) were identified downstream of *bla*_OXA-58_ in all isolates. However, the *araC1* and *lysE* in structure B were disrupted by IS*Aba20* and Tn*aphA6* respectively. IS*Aba20* is a novel insertion sequence of IS*3* family, and is 1199 bp long with two ORFs. The insertion of IS*Aba*20 into *araC1* generated two 4-bp direct repeats (CTTA). Tn*aphA6* was a composite transposon, comprising an aminoglycoside *O*-phosphotransferase gene, *aphA6*, and two flanked IS*Aba125* of same orientation. Tn*aphA6* was inserted into *lysE* and generated two 3-bp target site duplications (CTG).

It has been reported that the acquisition of *bla*_OXA-58_ is usually associated with recombination events characterized by the presence of two 27-bp sequences named Re27-1 and Re27-2 [9]. In structure A, we identified a similar Re27-1 sequence located 8 bp downstream of the intact IS*Aba3*-like element (5′-ATTTAACATAATGGCTGTTATACGAAA-3′), and an imperfect Re27-2 sequence (5′-ATTTAACATAATGGTGGTTATACGCAA-3′) was just adjacent to the downstream of *lysE*. In structure D, a pair of 29-bp imperfect probable recombination points were identified 748 bp downstream of IS*15* element (5′-ATTTAACATAATGGTGGTTATGCGAAGTC-3′) and adjacent to *lysE* (5′-ATTTAACATAATGGGCGTTATGCGAAGTC-3′). In structure B and C, we failed to find pairs of Re27-like regions.

## Discussion

Previous studies reported that European clone II lineage OXA-23-producing *A. baumannii* CC92 was the most popular carbapenem-resistant clone in China [24], [27]. The OXA-58 producing *A. baumannii* of European clone II has been reported in Italy [28], Greece [29] and China [30]. However, only *A. baumannii* ST91 and ST363 were identified in this study without any European clone II lineage isolates. We have showed ST91 strains contain both *bla*_OXA-23_ and *bla*_OXA-58_, and possess multidrug resistance to carbapenems, broad-spectrum cephalosporins, aminoglycosides, ampicillin/sulbactam, minocycline, and ciprofloxacin. Moreover, ST91 was detected in two cities. Therefore, we speculate ST91 is a potential risk multidrug resistant clone that is widely present in China. A larger scale epidemiological investigation would be necessary to fully elucidate the true distribution of ST91 in China.

Gentamicin and amikacin resistance were observed in transformants of *A. baumannii* ST91. The analysis of nucleotide sequence around *bla*_OXA-58_ identified an aminoglycoside *O*-phosphotransferase gene, *aphA6*. The gentamicin and amikacin resistance gene *aphA6* was first reported in *A. baumannii* in 1988 [31]. Nigro et al. recently reported *aphA6* located in a potential transposon Tn*aphA6*, flanked by two copies of IS*Aba125* [32]. An identical transposon was identified in our study and Tn*aphA6* was inserted into a putative threonine efflux protein gene *lysE*. It should be noticed that the susceptible *A. baumannii* could develop carbapenem and amikacin resistance simultaneously via the *bla*_OXA-58_ and *aphA6* co-harboring plasmid.

Bertini et al. reported the *bla*_OXA-58_ harboring plasmids could be classified into various groups, including GR2 (Aci1), GR3 (Aci3 and Aci7), GR4 (Aci4) and GR5 (Aci5) [26]. Using the same typing scheme, Towner reported that OXA-58 producing *A. baumannii* from European countries were commonly associated with Aci1, Aci3, Aci4, and AciX [15]. However, the *bla*_OXA-58_-carrying plasmids in this study did not belong to any known replicon groups, and a novel replicase gene *rep*Aci10 was identified This suggests that the spread of *bla*_OXA-58_ in China may be mediated by unique plasmids being different from those of Europe. Meanwhile, the plasmids of *rep*Aci10 are viable in different genomic species of *Acinetobacter* and may contribute to horizontal transmission of resistance genes. However, the replicase genes of the plasmids of *A. baumannii* ST91 remain unknown and further complete plasmid sequencing is in process.

The acquisition of *bla*_OXA-58_ is associated with a recombination event at site of Re27 sequence [9]. However, the pairs of Re27 sequence around *bla*_OXA-58_ were absent in partial isolates in this study, suggesting it may have been lost during plasmid evolution.

It is speculated that the insertion of other IS element into IS*Aba3*-like could generate a hybrid promoter to enhance the transcription of *bla*_OXA-58_ and mediate greater carbapenem resistance than the intact IS*Aba3*-like element as previously reported [9]–[11], [33]. In this study, for plasmids that the IS*Aba3*-like element was disrupted by IS*Our1*, IS*1008* or IS*15*, their corresponding transformants showed a high increase in imipenem MICs (16–32 folds), while for plasmids that the IS*Aba3*-like element was intact, the imipenem MICs of their corresponding transformants were only slightly increasing (2–4 folds). The special structure of IS*6* family-ΔIS*Aba3*-like-*bla*_OXA-58_ is different from IS*Aba2*, IS*18*, IS*Aba125*, IS*Aba1* and IS*Aba825* that is usually inserted into IS*Aba3*-like in *Acinetobacter* spp. from Europe [9], [34], [35].

In conclusion, the genetic background of OXA-58-producing *Acinetobacter* spp. in China was diverse, and the multidrug resistant *A. baumannii* ST91 is a potential risk clone. The STs of *A. baumannii*, replicon typing of *bla*_OXA-58_-harboring plasmids and genetic contexts of *bla*_OXA-58_ were distinct from those of Europe, implying the unique evolution and transmission pattern of *bla*_OXA-58_ in *Acinetobacter* spp. in China.
